# Phenyliminophenothiazine based self-organization of polyaniline nanowires and application as redox probe in electrochemical sensors

**DOI:** 10.1038/s41598-018-36937-5

**Published:** 2019-01-23

**Authors:** Alena I. Khadieva, Vladimir V. Gorbachuk, Gennady A. Evtugyn, Svetlana V. Belyakova, Ruslan R. Latypov, Sergey V. Drobyshev, Ivan I. Stoikov

**Affiliations:** 1 A.M. Butlerov Institute of Chemistry of Kazan Federal University, 420008, Kremlevskaya, 18, Kazan, 420008 Russian Federation; 2 Institute of Physics of Kazan Federal University, 420008, Kremlevskaya, 18, Kazan, 420008 Russian Federation; 3 Kazan National Research Technical University named after A. N. Tupolev – KAI, K. Marx Street, 10, Kazan, 420111 Russian Federation

## Abstract

Synthesis and application of nanostructured materials applicable in the assembly of electrochemical sensors is one of the important trends in material sciences and analytical chemistry. In this work, we have proposed and implemented simple non-template method for assembling nanofibers from the polyaniline ultrasonicated with phenyliminophenothiazine in aqueous media. Two-step procedure including association with emeraldine dispersion and reorganization under ultrasonication led to formation of nanofibrillar structures with average diameter of 20 nm. UV-spectroscopy confirms that association of phenyliminophenothiazine and polyaniline in acidic medium resulted in an intense absorption band at 900–910 nm due to donor-acceptor interaction between the reactants. The material combined emeraldine charge transmission with redox activity of phenyliminophenothiazine was found promising for electrochemical sensing. It was confirmed by comparison of characteristics of appropriate solid-contact sensors based on emeraldine and phenyliminophenothiazine toward Fe(III) ions, ascorbic acid and hydroquinone. In all the cases, the use of phenyliminophenothiazine results in a wider concentration range and more reproducible signal against characteristics of similar sensor based on polyaniline. The applicability of the sensor was confirmed by determination of iron content in commercial medication.

## Introduction

Growing technological applications of electroactive polymers keep the development and improvement of appropriate materials as one of the most important challenges. Nanofibers preparation based on semiconductors and organic materials with high electroconductivity is one of burning trends of modern material sciences. Such one-dimensional nanostructures are widely used in electroactive composite materials due to high specific surface area, stability in colloid systems and ability to form extended structures^1^. These properties are demanded in sensor development and in nanophotonics as elements commensurate with the wavelength of a visible light^2^. They are also applicable as nanosized analogs of metallic conductors of macroscopic instruments. Large surface area maintains an interest in such materials in gas sensing^3^ and liquids analysis^4^. Among variety of such materials, emeraldine, a semi-oxidized form of polyaniline, is most attractive due to easy preparation, low toxicity and low electric resistance^5^. These advantages led to a number of additional applications in biointerfaces. Various approaches have been described at the moment for the synthesis of polymeric nanostructures, e.g., electrospinning, soft- and hard-template synthesis, sonochemical methods^6^, interfacial and non-agitated oxidative polymerization, etc.^7^ All these approaches have certain advantages. Thus, porous organized solid templates allow producing aligned nanofibers demanded in optics. Nanofiber preparation in solution is more convenient for assembling of sensors and nanocomposites. Regarding emeraldine, oxidative bulk polymerization is mostly mentioned though it is rather complex and difficult for scaling up.

Redox-active polymers have found increasing application in electrochemical sensors as signal transducers^8^. Thus, polyaniline, both chemically and electrochemically polymerized, was used in the assembly of potentiometric sensors for the determination of metal ions^9–11^. The necessity in such materials is explained by reversibility of their redox reactions coupled with the hydrogen ions transfer^12^. This makes it possible to exclude internal filling from the assembly of potentiometric sensor and prepare miniaturized devices with arbitrary size and shape. However, the number of materials appropriate for assembling of these solid-contact sensors as well as variety of analytes determined are rather limited. Thus, few reports are devoted to the copolymers of aniline with other aromatic amines that show reversible signal and sensitivity of the potential toward the ionic content of the media^13–15^.

In this work, we propose a simple one-stage method for the preparation of polyaniline nanofibers by conventional oxidative polymerization, which provides self-assembling of polyaniline nanofibers by sonication of the associates formed by emeraldine and phenyliminophenothiazine (PTZANI). Previously it was shown in the literature that monomeric aromatic amines^16–18^, phenol and hydroquinone^19^ affect the emeraldine morphology on the stage of its oxidative polymerization. Phenol can also promote solubilization of the polymerization products in aqueous media. In this work, another aromatic additive, PTZANI, was for the first time used for reorganization of dispersed polyaniline into nanofibers. The nanofibers obtained exerted a high absorption band in near IR region (900–910 nm) attributed to the donor-acceptor interaction with the charge transfer. It was shown on the example of model antioxidants and Fe(III) ions that PTZANI can be utilized as sensing element in the electrochemical sensors intended for monitoring redox properties of various species.

## Experimental

### Materials

Ammonium persulfate, *p*-toluenesulfonic acid, ascorbic acid, hydroquinone and phenothiazine were purchased from Sigma-Aldrich and used as received without additional purification. Carbon black was purchased from Cabot Norit b.v. (the Netherlands). Aniline (Sigma-Aldrich) freshly distilled under vacuum was used for the synthesis. Other chemicals were of reagent grade. Organic solvents (ethanol, dichloromethane, propanol-2, tetrahydrofuran (THF)) were purified using common laboratory protocols. Ferrum Lek ® tablets (Lek, Slovenia) were used as a source of iron.

## Materials and Methods

NMR ^1^Н и ^13^С spectra were recorded with the Bruker Avance 400 spectrometer (101 and 400 MHz, respectively). Chemical shifts were determined against the signals of residual protons of deuterated dimethylsulfoxide (CD_3_)_2_SO. UV-Vis-NIR-spectra were recorded in 1 cm quartz cuvettes with Shimadzu UV-3600 spectrometer in distilled water. Concentration of the sample solutions was equal to 1 mM in these experiments. Gas chromatography–mass spectrometry (GC–MS) data was obtained on Shimadzu GCMS-QP2010 with 70 eV electron impact and direct probe inlet using dichloromethane as eluent. Size distribution of the particles and the polydispersity index (PdI) were measured in 1 mM aqueous dispersion by dynamic light scattering (DLS) with the particle size analyzer Zetasizer Nano ZS (Malvern, Great Britain). Measurements were carried out at 173° detection angle. The PdI for colloid systems was calculated as r^2^/Z_D_^2^, where r is standard deviation (SD) of the hypothetical Gaussian distribution and Z_D_ the Z average size, the intensity-weighted mean hydrodynamic size. Samples with PdI ≤ 0.2 were considered as monodispersed^20^. The PdI > 0.7 value was considered as an evidence of a broad size distribution not suitable for the following consideration in the DLS technique^21^. Elemental analysis was performed with the Perkin Elmer 2400 Series II Instrument. Purity of reagents and the reaction course were monitored by the thin-layer chromatography on the Silica G plates, 200 µm, UV 254. Ultrasound dispergation was performed with the Sonics Vibracell VCX 500. Scanning electronic microscopy (SEM) imaging was carried out with the Carl Zeiss Auriga Cross Beam microscope on the aluminum foil. Colloidal samples were diluted with propanol-2 to final concentration of 1 × 10^−4^ g/ml, poured onto the aluminum foil and left to dry in vacuum desiccator for one hour.

### Synthesis of (E)-N-phenyl-3H-phenothiazin-3-imine (PTZANI)

Aniline (0.46 g, 5.0 mmol), phenothiazine (1.0 g, 5.0 mmol), and *p*-toluenesulfonic acid (1.9 g, 10.0 mmol) were dissolved in 50 ml of THF at ambient temperature. The reaction mixture was stirred for one hour. Ammonium persulfate solution in distilled water was dropwise added to the reaction mixture within 30 min. Then the reaction mixture was stirred for 48 h and neutralized with 2% aqueous ammonia. The sedimented product was separated by filtration and purified in the Soxhlet extractor with ethanol. Purple powder was collected and dried overnight in vacuum desiccator over P_4_O_10_ (yield 70%).

^1^H NMR (400 MHz, (CD_3_)_2_SO): δ 7.69 – 7.60 (m, 1H), 7.43 (m, 6H), 7.17 (dd, 2H), 7.06–6.63 (m, 4H).

^13^C NMR (101 MHz, (CD_3_)_2_SO): δ 138.8, 136.7, 135.6, 132.8, 132.7, 130.8, 130.7, 129.7, 129.6, 128.4, 128.3, 126.3, 125.9, 125.8, 124.9, 123.9, 120.7, 112.4.

Elemental analysis: calculated for C_18_H_12_N_2_S: C, 74.97; H, 4.19; N, 9.71; S, 11.12, experiment: C, 75.01; H, 4.17; N, 9.65; S, 11.09.

GC–MS: (M+) 290 (retention time 30.09 min, Supelco column).

### Oxidative polymerization of aniline

Aniline (0.93 g, 10.0 mmol) and *p*-toluenesulfonic acid (1.9 g, 10.0 mmol) were dissolved in 50 ml of distilled water at ambient temperature. Then the solution of ammonium persulfate (0.57 g, 2.5 mmol) in 50 ml of distilled water was added under fast stirring (400 rpm) to the reaction mixture. Stirring was stopped after 1 min and the reaction mixture was left for 24 hours with no agitation. The green precipitate obtained was separated by filtration and then dried overnight in vacuum desiccator over P_4_O_10_ (yield 70%).

^1^H NMR (400 MHz, (CD_3_)_2_SO): δ 2.28 s (proton signals of CH_3_ of *p*-toluenesulfonic acid), 7.1 m, 7.5 m (proton signals of Ar-H of *p*-toluenesulfonic acid), 6.9–7.8 m (Ar-H)

^13^C NMR (101 MHz, (CD_3_)_2_SO): δ 115.0, 118.0, 121.0, 124.0.

### Preparation of the PTZANI /polyaniline mixture

Mixture of PTZANI (1.45 g, 5 mmol), emeraldine (3.74 g, 5 mmol) and *p*-toluenesulfonic acid (1.9 g, 10 mmol) was dissolved in 50 ml of distilled water and left under magnetic stirring for 24 h. The dark blue-green precipitate obtained was collected and then dried overnight under vacuum (yield 90%). For preparation of the nanofibers, the reaction mixture of the same content was stirred and ultrasonicated for 24 hours during which its color was changed from dark blue to black. The precipitate was separated by filtration and dried overnight under vacuum (yield 90%).

### Electrochemical measurements

All the measurements were performed in two-electrode cell with the Ag/AgCl reference electrode. Planar carbon electrode prepared by screen-printing was modified with the redox active materials and used as a working electrode. Screen-printed electrodes were produced on polycarbonate film with DEK-248 screen-printing machine (DEK, England). Electroconductive silver tracks, carbon paste and insulator layers (Gwent, United Kingdom) were consecutively deposited on the polycarbonate film using templates and thermally hardened at 80 °C.

The pH and potential measurements were performed with the digital pH-meter Expert-001 (Econix Expert, Russia). Voltammetric measurements were performed in three-electrode non-thermostated cell with Autolab PGSTAT 302 N potentiostat (Metrohm Autolab, the Netherlands) using the same working and reference electrode and Pt wire as counter electrode.

### Modification of screen-printed electrode

First, carbon black was sonicated in dimethylformamide (DMF) and 2 μl of dispersion were placed on the working surface of the electrode and dried for 20 min at 60 °C. Then, the aliquot of 300 or 600 μl (24 and 48 μmol, respectively) of 0.08 M solution of PTZANI in chloroform was evenly distributed on the surface and dried at ambient temperature. After that, 3 or 6 μl of the PTZANI chloroform solution (final content of 24 or 48 μmol per electrode) were placed on the working area of screen-printed electrode and left to dry until formation of the colored dense film.

### Measurement protocols

In potentiometric measurements, the working and reference electrodes were immersed in the cell containing 20 ml of 0.01 M nitric acid and conditioned under stirring with magnetic stirrer until a stable potential with the drift less than 0.1 mV/min was reached. Voltammetric measurements were performed in direct current mode in the same solution by scanning the potential from −0.4 to + 0.8 V in the solution of 1.0 × 10^−2^ M K_3_Fe(CN)_6_ with the scan rate of 50 mV/s. The heterogeneous rate constant *k*° of the electron transfer was calculated using Klinger-Kochi method^22^ (1)

1 $${k}^{0}=2.18{[\frac{\alpha DnFv}{RT}]}^{1/2}\exp [-\frac{{\alpha }^{2}nF}{RT}({E}_{p}^{a}-{E}_{p}^{c})],$$

where α is the transfer coefficient determined from the Nicholson equation^23^ (3), *D* is diffusion coefficient (*D* = 7.2 × 10^−6^ cm/s for ferricyanide ion^24^), *n* is the number of electrons transferred (*n* = 1), ν is the scan rate, ${E}_{p}^{a},{E}_{p}^{c}$ are anodic and cathodic peak potentials, respectively. Three replications with freshly prepared modified electrodes were used.

2 $$\alpha =\frac{{E}^{0}-{E}_{p}^{c}}{{E}_{p}^{a}-{E}_{p}^{c}},$$

where *E*^0^ is the formal redox potential of [Fe(CN)_6_]^3−/4−^ pair. For antioxidant measurements, working solutions of ascorbic acid, hydroquinone and K_3_Fe(CN)_6_ were prepared by dilution of their stock solution freshly prepared in water. No pH correction was made.

### The determination of iron content in Ferrum Lek^®^ medication

Screen-printed electrode modified with carbon black and PTZANI (48 μmol) was immersed together with Ag/AgCl reference electrode in the working cell containing 20 ml of 0.1 mM nitric acid and then its potential was measured with digital ionometer after reaching its stationary value (within 10 min). Medication sample was grinded and then dissolved in nitric acid. The solution was filtered and then diluted to approx. 12 mM Fe(III) concentration, mixed in 1:1 vol. ratio with 50% HNO_3_ and evaporated on the water bath. After that, 10 ml of distilled water were added and resulting volume adjusted to 25 ml with 10 mM nitric acid.

## Results and Discussion

### Synthesis of PTZANI

**Figure 1:**
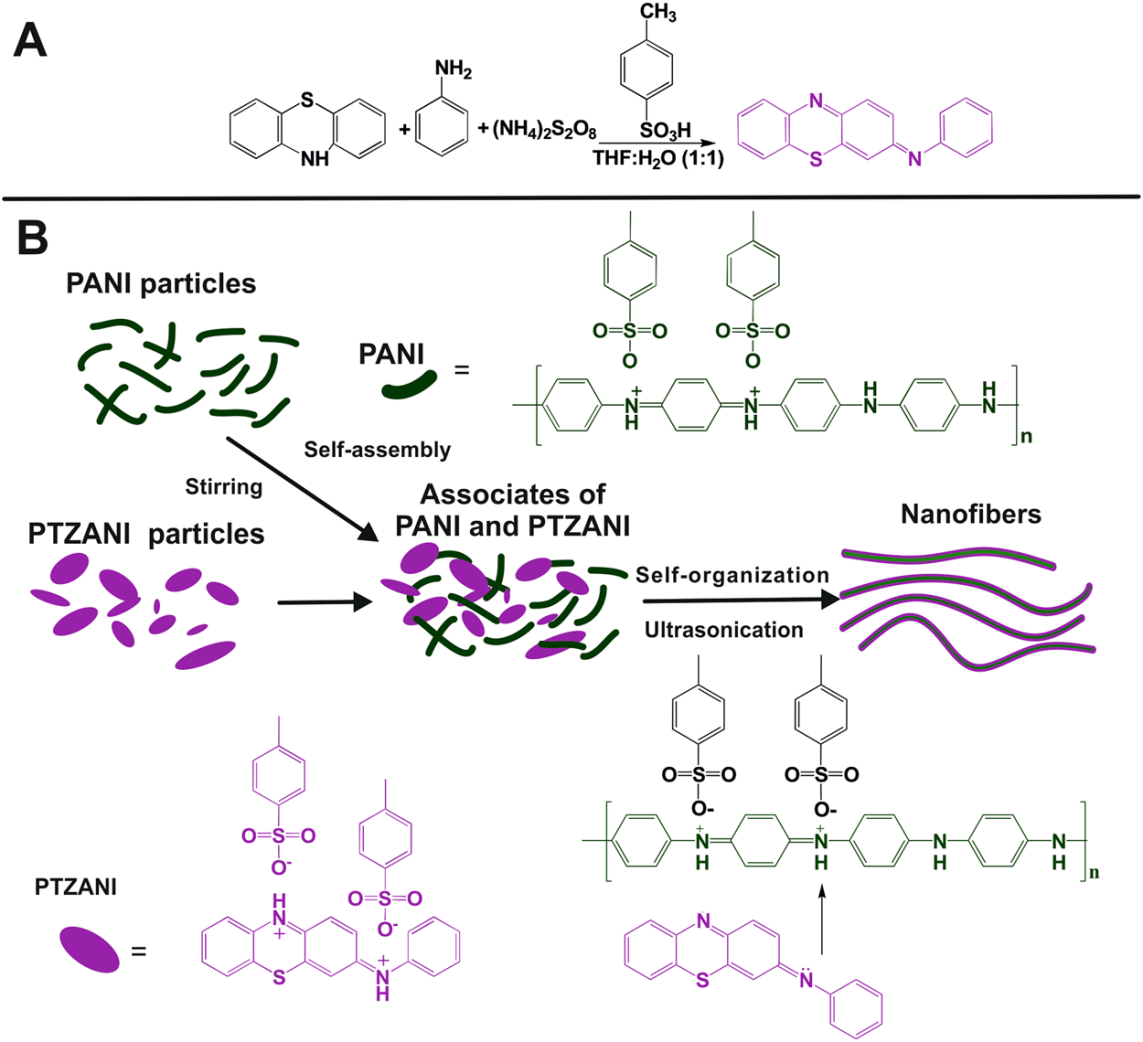
(**A**) Preparation of PTZANI by oxidation of a mixture of phenothiazine and polyaniline (PANI) and (**B**) schematic flow chart about the synthesis of nanofibers.

Only one method based on iron salt application was reported in the literature for the PTZANI synthesis. It included complicated separation of target compound from by-products^25–27^. We propose a simple, one-step protocol for the PTZANI preparation by oxidation of the mixture of phenothiazine and aniline with an excess of ammonium persulfate (Fig. 1A). The reaction of oxidative coupling of aniline and phenothiazine in the 1:1 ratio was carried out in homogeneous conditions in the THF/water mixture (1:1 v/v) at ambient temperature. The *p*-toluenesulfonic acid and ammonium persulfate were added to convert arylamines into corresponding salts and their oxidation, respectively. The formation of the target product, PTZANI, in the above reaction was confirmed by GC–MS using the reaction mixture washed with aqueous ammonia and water to remove excess of *p-*toluenesulfonic acid and inorganic impurities (Supporting Information). Small amount (4%) of isomers (30.2 min, 31.8 min, 33.3 min) and of the starting compound (phenothiazine, 13.9 min) were found on chromatograms together with the main product of the N-C coupling (PTZANI) which yield exceeded 96% according to the integrated intensities. Recrystallization in an aqueous ethanol made it possible to separate the product formed by coupling aniline to the 3-position of 3H-phenothiazine (against N - atom of phenothiazine) with 90% yield.

### Synthesis of nanofibers: self-organization of PTZANI and polyaniline

The formation of the polyaniline nanostructures is commonly achieved by the variation of the reaction conditions (application of templates, surfactants, sonication of the mixture) at the stage of oxidative polymerization^28,29^. It is known that some aromatic compounds, e.g. phenol, tetracyanoquinone, can interact with emeraldine chain and improve solubilization of the polymer. We supposed that interaction between the dispersion of emeraldine salt and PTZANI could result in formation of the charge-transfer complex via interaction of aniline fragments with electron-rich aryl groups of PTZANI and that such an interaction would affect morphology of the product. To prove this hypothesis, the molar ratio of emeraldine (tetraaniline fragment) and PTZANI in reaction medium was chosen to be 1:1. The reorganization of polyaniline and PTZANI associates into the nanofibers was promoted by their ultrasonication and studied by UV spectroscopy, DLS and SEM in comparison with the mixture of PTZANI and polyaniline dispersions taken with no ultrasonication.

### UV-Vis Investigation of the interaction between PTZANI and polyaniline

**Figure 2:**
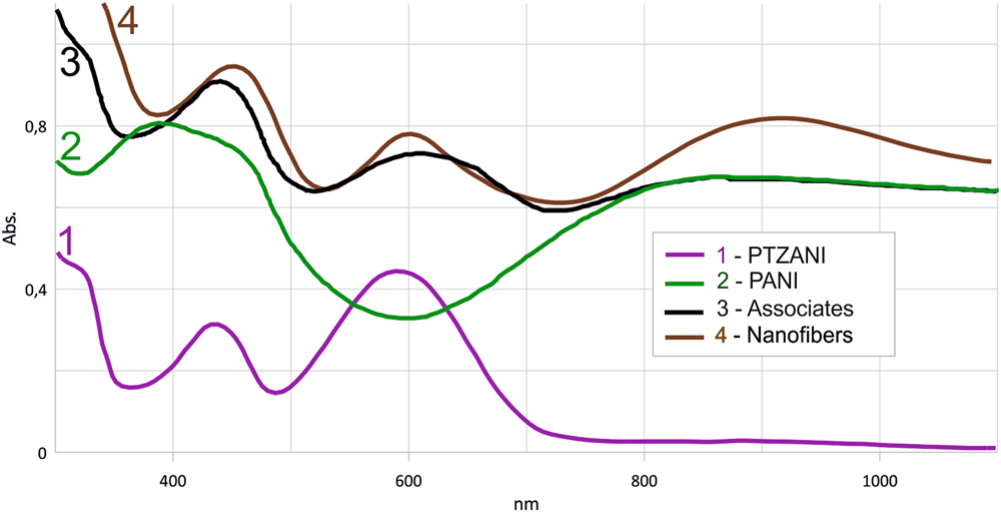
UV-VIS-NIR spectra of dispersions in water in the presence of *p*-toluenesulfonic acid (1 mM, at room temperature): PTZANI (1), emeraldine (2), mixtures of emeraldine and PTZANI before (3) and after (4) ultrasonication.

An intense absorption in the near IR region was reported for imino derivatives of phenothiazine. This made them very promising for application in the photothermal therapy^30^. We investigated spectral properties of self-organization products of PTZANI and polyaniline. Four aqueous dispersions were obtained in the presence of *p*-toluenesulfonic acid, polyaniline, PTZANI and their mixtures and investigated before and after ultrasonication by UV-VIS-NIR spectroscopy (Fig. 2).

For PTZANI, intense absorption peak at 275 nm corresponds to n-p* junctions. In the presence of *p-*TsOH, intense absorption band at 590–630 nm appears. After PANI and PTZANI mixing, no new absorption bands appear. After ultrasonication of the mixture, absorption was observed with the maximum at 900–910 nm. This band could not be attributed to any primary reagents and indicated the formation of associates between emeraldine and PTZANI (Fig. 1B).

Investigation of the interaction between PTZANI and polyaniline promoted by ultrasonication and of the formation of nanofibers was further carried out by DLS and SEM.

### DLS investigation of nanofibers self-assembled from the PTZANI and polyaniline

**Figure 3:**
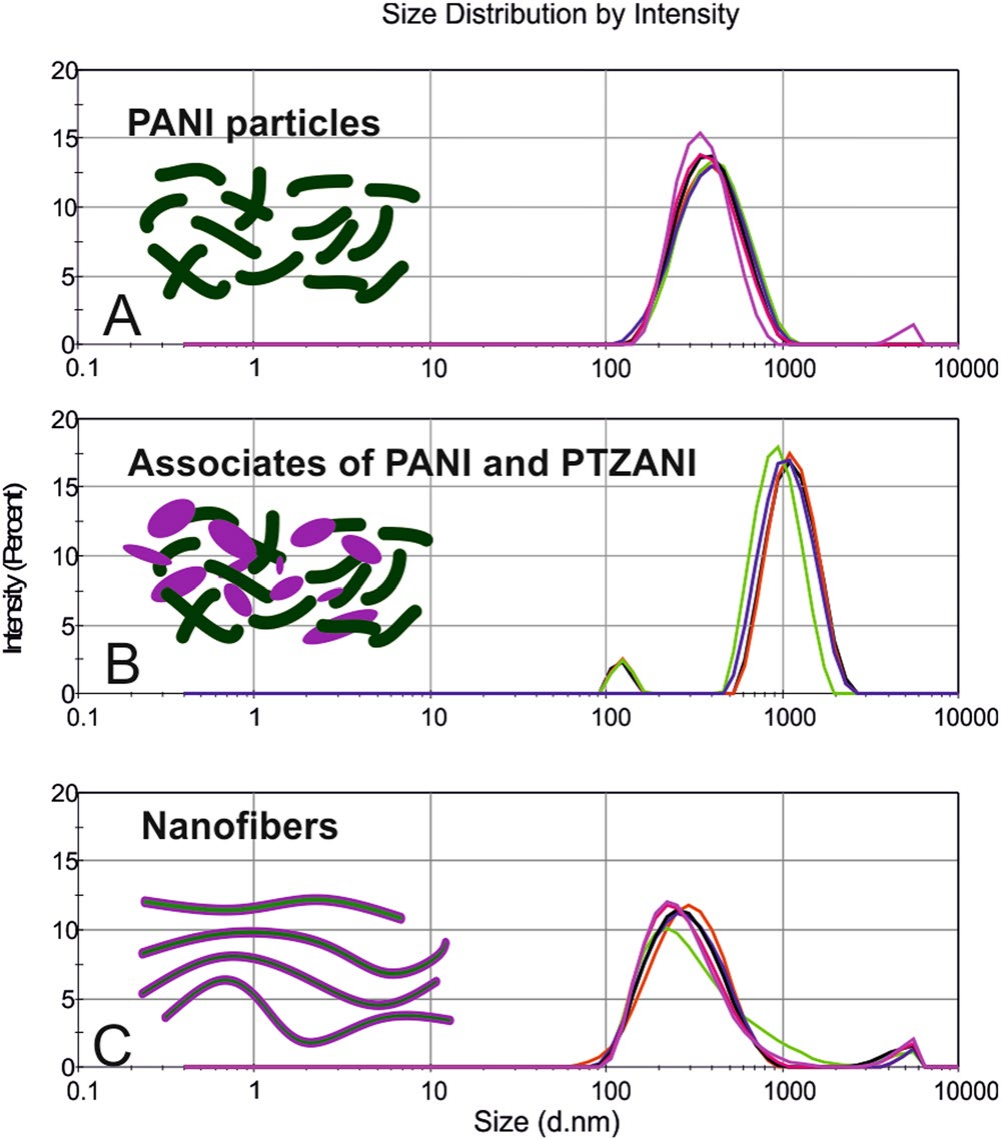
Size distribution by intensity in water in the presence of *p*-toluenesulfonic acid (1 mM): (**A**) emeraldine (1 mM), mixture of emeraldine and PTZANI (1 mM) before (**B**) and after (**C**) ultrasonication.

The associates of PTZANI and polyaniline obtained by stirring form in water particles with the average size of 949 nm and PdI = 0.3. The same associates obtained under ultrasonication showed the average size of 278 nm and PdI = 0.2 (Fig. 3). Thus, continuous ultrasonication led to self-organization associates with smaller size in submicron range and lower PdI. This was considered as an evidence of aggregation of PTZANI with the polyaniline particles.

### SEM investigation of the morphology of the mixture and nanofibers of PTZANI and polyaniline

**Figure 4:**
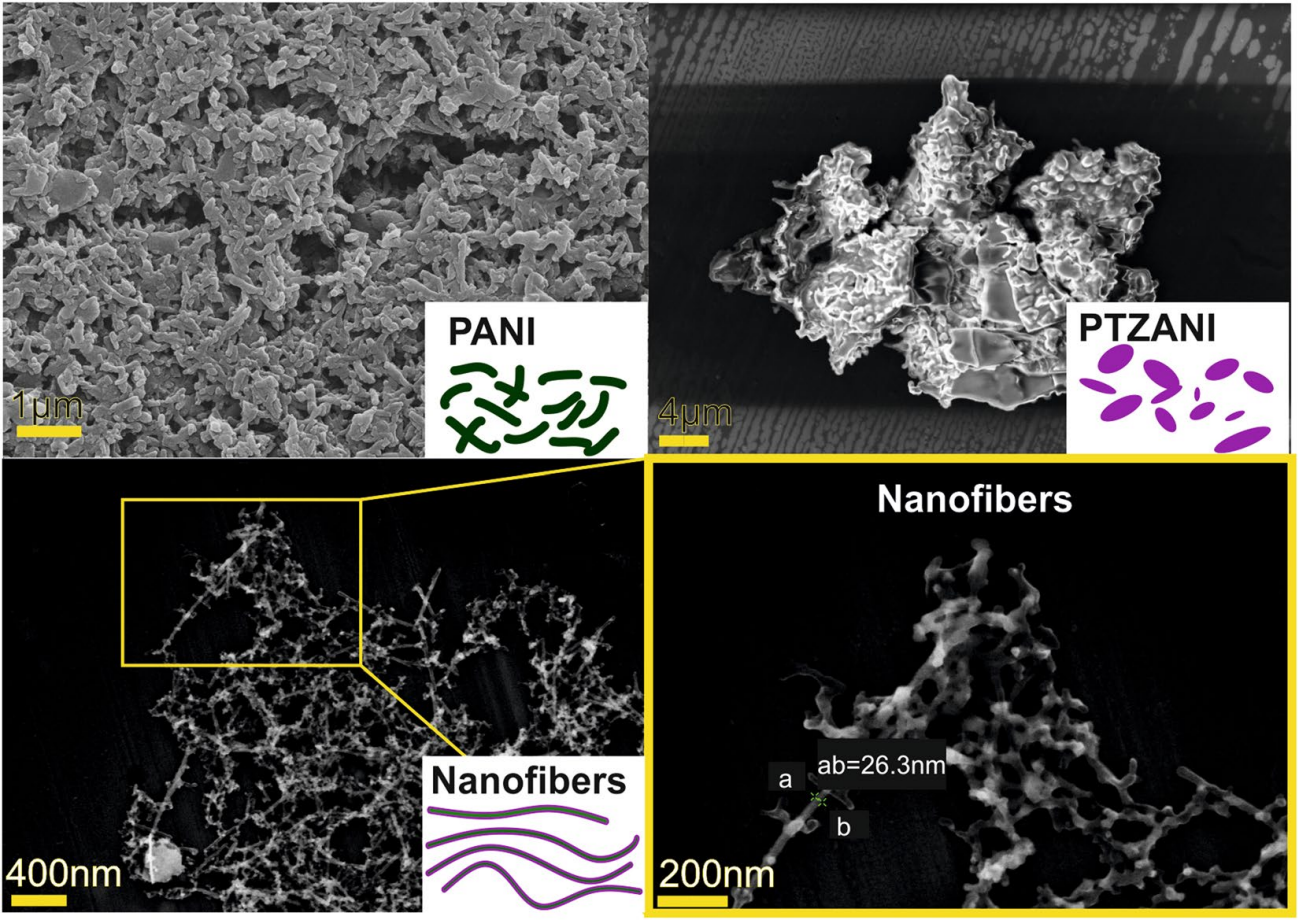
SEM images of (**a**) emeraldine (**b**) mixture of emeraldine and PTZANI obtained by stirring (**c**,**d**) nanofibers of emeraldine and PTZANI obtained by ultrasonication

**Figure 5:**
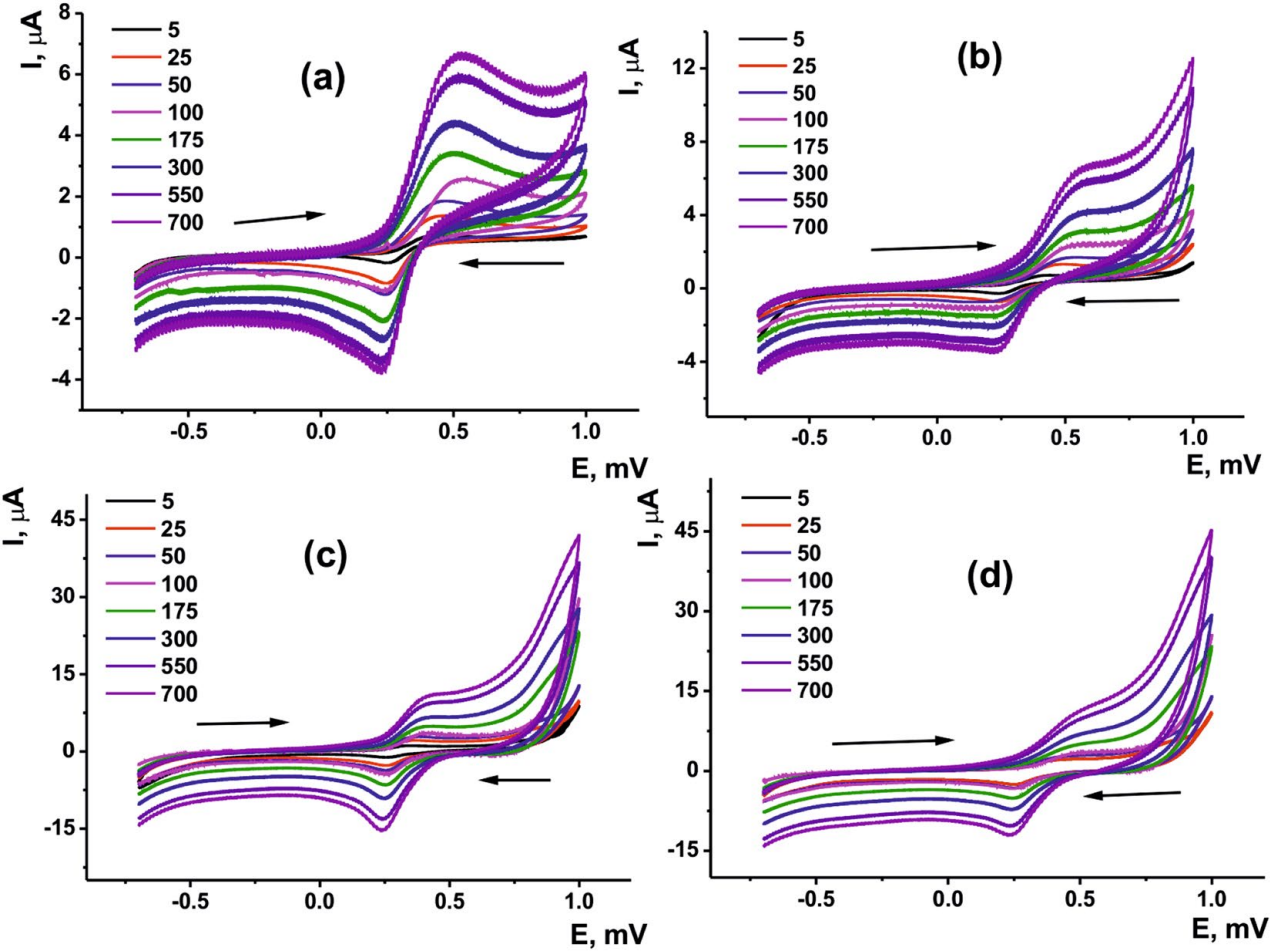
Cyclic voltammograms recorded at various scan rates in the 5 × 10^−4^ М solution of ferricyanide ions on the screen-printed carbon electrodes covered with 24 μmol (**a**,**c**) and 48 μmol (**b**,**d**) PTZANI. Measurements on bare (**a**,**b**) and carbon black covered (**c**,**d**) electrodes.

Three dispersions in water were considered, i.e., that of polyaniline (Fig. 4a), of the mixture of polyaniline and PTZANI obtained under stirring (Fig. 4b), and that of nanofibers of polyaniline and PTZANI obtained under ultrasonication (Fig. 5c,d). One could see, stirring of the mixture of PTZANI and polyaniline led to formation of aggregates, where the surface of the polyaniline particles was coated with the PTZANI (Fig. 4b). Continuous ultrasonication of the dispersion of PTZANI and polyaniline resulted in color change from dark-green to black. SEM images showed nanofibers with average size of about 30 nm (Fig. 5d). SEM results are in agreement with the DLS data previously described and confirmed aggregation process. Average size of the polyaniline particles was estimated as 300 nm (SEM) and 371 nm (DLS, PdI = 0.2). The size of the associates formed from the mixture of PTZANI and polyaniline was estimated as 949 nm (DLS, PdI = 0.3) and that of several microns after the solvent removal. Latter result indicates further aggregation of the PTZANI and polyaniline.

Summarizing the SEM and DLS results, continuous ultrasonication led to reorganization of associates of emeraldine salt with PTZANI followed by their self-organization to submicron associates (278 nm, PdI = 0.2) in the form of nanofibers with length of 200–600 nm and diameter of 20–30 nm.

In the literature, disaggregation of emeraldine was mostly followed by size exclusion chromatography. Its dispersion in *N*-methylpyrrolidone led to lower size of the particles. The process was accelerated by lithium salts or ionic liquids addition^31^. In aqueous solutions, stabilization of emeraldine particles with poly (*N*-vinylpyrrolidone) was reported^32^. In this work, similar effect of PTZANI explained by intermolecular interactions has been for the first time described. Significant morphological changes observed were stimulated by two factors, i.e., structural similarity of the PTZANI and emeraldine units and charge transfer complex formation.

### Application of PTZANI in potentiometric sensors

Both PTZANI as phenothiazine derivative and emeraldine should exert reversible redox conversion at appropriate potentials. Being pH-sensitive, redox equilibria result in possibility to measure species that either change the charge of the layers or redox potential of the environment. Polyaniline based sensors are known in the assembly of solid-contact potentiometric sensors described in the literature for metal ions detection^10−12,14,15^. Structural similarity of emeraldine trimer and PTZANI and their ability to form composite nanofibers can significantly alter their redox activity. For this reason, it was interesting to compare the performance of solid-contact potentiometric sensors obtained from PTZANI or emeraldine separately deposited on the screen-printed electrode. Three redox active substrates different in charge and redox properties, i.e., hydroquinone, ascorbic acid, and Fe(III) ions, were chosen for comparison.

Deposition of sensing layer was performed by drop-casting of the suspensions containing carbon black as support providing mechanical durability of the layer and better reproducibility of the sensor characteristics. Prior to potentiometric measurements, redox reversibility of the modified electrode was estimated using ferricyanide ion as redox probe with direct current voltammetry (Fig. 5). A reversible peak pair was observed on cyclic voltammograms in accordance with the transfer of one electron. Ratio of the cathodic and anodic peak currents tends to 1. Together with symmetrical shape of the peaks and small peak potential difference this confirms high rate of the electron exchange and suitability of the modified electrode for the application in the assembly of potentiometric sensor. The parameters of the electron transfer, i.e., heterogeneous rate constant *k*^0^ and transfer coefficient α determined by Klinger-Kochi and Nicholson methods, respectively, are summarized in Table 1 for three replications made with freshly prepared electrodes, standard deviation of the above parameters did not exceed 5%.

**Table 1:** Heterogeneous rate constants of electron transfer *k*^0^ × 10^3^ cm/s and transfer coefficient α depending on the modification of bare (a,b) and carbon black covered (c,d) screen-printed electrode with 24 (a,c) and 48 μmol (b,d) of PTZANI. Scan rate Measurements on bare (a,b) and carbon black covered (c,d) electrodes.

Layer content	a	b	c	d
k^0^ × 10^3^, cm/s	2.56	1.92	2.73	2.62
α	0.20	0.25	0.34	0.39

As could be seen, all the modification protocols provide a high rate of the electron transfer indicating their applicability as transducers of potentiometric sensors measuring redox potential of the environment. In the presence of carbon black, both the *k*^0^ and α values increase against bare electrode due to higher active surface of electrodes and better interaction with the polymers casted due to their electrostatic attraction. Meanwhile the transfer coefficient remains below theoretic value of 0.5 corresponded to true redox equilibrium on the electrode interface. This might be due to negative charge of the electrode support caused by carboxylic groups placed on the surface of carbon paste of the electrode layer and carbon black particles. This means preferable existence of the reduced form [Fe(CN)_6_]^4−^ over its equilibrium value and hence potentially higher sensitivity of the electrode potential toward oxidants present in the sample tested.

The following experiments were performed in open circuit mode with no ferricyanide ions in the solution.

The potentiometric sensor exerted stable well reproducible potential with no respect of carbon black deposition and quantified of PTZANI added (24 or 48 μmol per electrode). Its potential depended on the pH value due to participation of the H^+^ ions in the redox conversion of the polymer. The appropriate dependence is linear in the pH range from 3.0 to 9.0 with the slope of 52–55 mV/pH indicating equal number of electrons and H^+^ ions transferred. The pH changes of the potential are fully reversible and did not depend on the direction of the pH shift (from acidic media to basic solution or vice versa). In case of carbon black deposition, the pH response becomes slightly lower in neutral and acidic media due to own buffering properties of the support caused by carboxylic groups of carbon particles. Besides, the addition of carbon black in the surface layer improved the repeatability of the potential (3.5 and 2.2% for six sensors with PTZANI on bare and carbon black modified electrode, respectively).

The ability of potentiometric sensors to monitor redox properties of the samples tested was confirmed by determination of Fe (III) ions and two antioxidants (ascorbic acid and hydroquinone) different in charge and oxidation mechanism. Ascorbic acid is irreversibly oxidized to dehydroascorbic acid whereas hydroquinone undergoes reversible two electron oxidation to benzoquinone.

**Figure 6:**
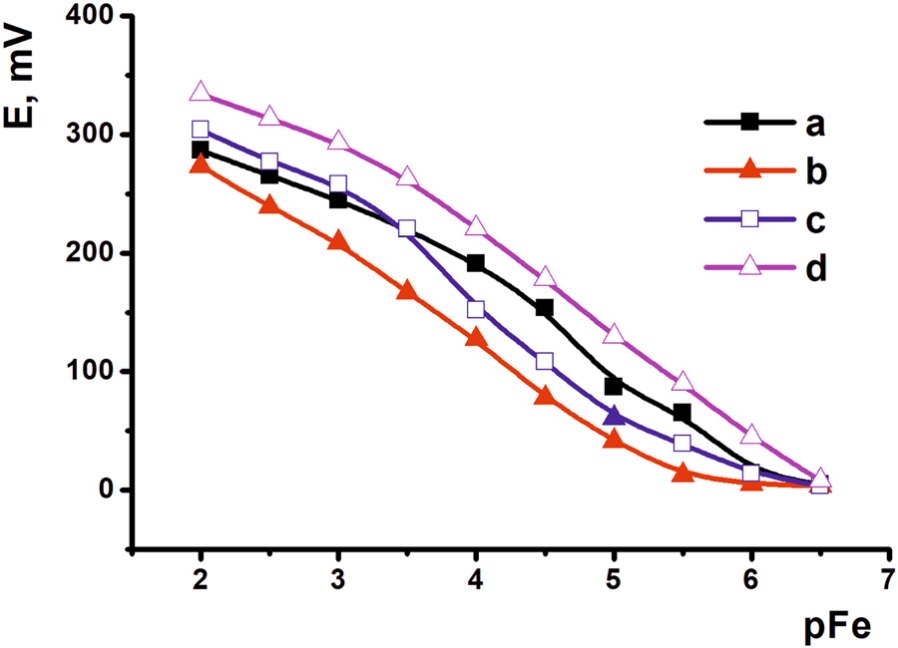
The dependence of the sensor potential on the Fe(III) ion concentration. The electrodes are covered with 24 μmol (**a**,**c**) and 48 μmol (**b**,**d**) PTZANI. Measurements on bare (**a**,**b**) and carbon black covered (**c**,**d**) electrodes.

Fe(III) ions calibration curve has a typical shape corresponding to the Nernst equation with a linear piece in the concentration range from 10^−2^ to 10^−6.5^ M (Fig. 6). Higher amount of the PTZANI added in the surface layer increases sensitivity of signal whereas carbon black improves reproducibility of the results. The analytical performance of the sensor is similar to that reported for Fe(III) determination with the ion-selective electrode made of chalcogenide glass^33^ and that with polymeric membrane containing benzyl thiocarbohydrazide ionophore (concentration range from 10^−2^ to 10^−7^ M^34^).

**Figure 7:**
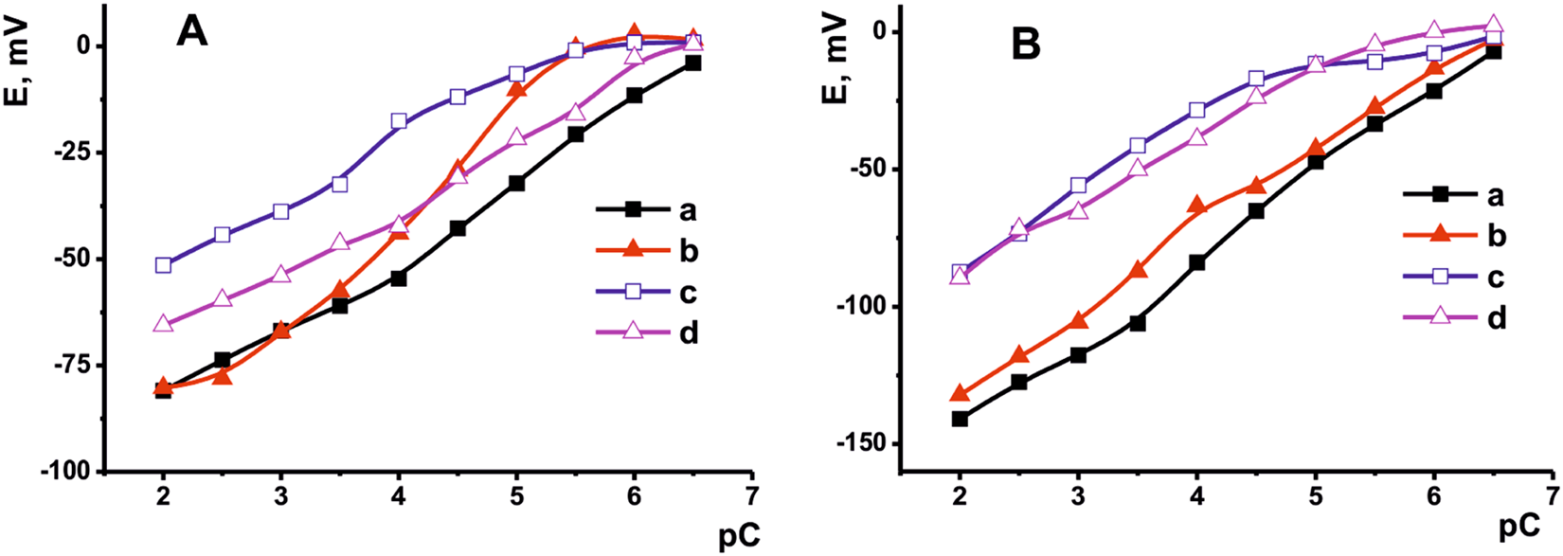
The dependence of the sensor potential on the hydroquinone (**A**) and ascorbic acid (**B**) concentration. The electrodes are covered with 24 μmol (a,c) and 48 μmol (b,d) PTZANI. Measurements on bare (a,b) and carbon black covered (c,d) electrodes.

It should be mentioned that introduction of polyaniline instead of PTZANI changes the calibration curve shape of Fe(III) ions to that typical for redox mechanism of the response. It has a super-Nernstian slope in a narrow range of concentrations with two sloping items in the area of small and high Fe(II) concentrations^35^. This is explained with direct oxidation of emeraldine with Fe(III) ions. In case of PTZANI, charge control of the potential prevails due to spatial separation of the Fe(III) ions and emeraldine chain by phenothiazine units coordinating the Fe(III) ion near thiazine fragment of the molecule. No other ions affect the signal of potentiometric sensor in a similar manner except Ag (I) and copper (II). However, their influence was found insignificant because of the lower ionic potential and lack of spatial complementarity to potential binding sites of the polymer. Taking into account simple preparation of the sensing layer and cost-effective protocol of screen-printing, such a behavior can be considered as important advantage of the potentiometric sensor with PTZANI layer over the analogs described in the literature. Similar experiments have been performed with hydroquinone and ascorbic acid (Fig. 7A,B, respectively). In case of hydroquinone, the slope of the calibration curve was about the same (17–22 mV/pC) for all the assemblies of the coating layer except that corresponded to higher PTZANI amount on bare screen-printed electrode. The latter one (29 mV/pC) corresponds to the transfer of one hydrogen ion and two electrons in the step limiting the potential of the sensor. The electrochemical sensor makes it possible to determine from 10^−6.5^ to 10^−2^ M hydroquinone with the limit of detection of 10^−7^ M. The limit of detection was calculated from S/N = 3 ratio. These characteristics exceed those reported for polyaniline based potentiometric sensor (concentration range 10^−4^–10^−2.4^ M, 25 mV/pC^35^). Calibration curve of ascorbic acid is influenced by the presence of carbon black and lesser depends on the PTZANI quantities. This might be due similar charge of the analyte and carboxylate groups of the carrier and hence to higher contribution of electrostatic interaction to the sensor potential. The calibration curve is linearized in the range from 10^−2^ to 10^−5^ M (bare screen-printed electrode) and from 10^−2^ to 10^−7^ M (carbon black covered electrode). The slope of the curves exceeds that of hydroquinone (30 mV/pC) and corresponds to transfer of two electrons and one hydrogen ion in the potential limiting stage of the reaction. In comparison, the polyaniline covered electrode provided linear response to hydroquinone in a very narrow range from 10^−4^ to 10^−3^ M and the slope of 28 mV/pC^35^. Thus, in both cases substitution of polyaniline with PTZANI improved the analytical characteristics of antioxidant determination. The measurement-to-measurement repeatability of the signal toward 10^−3^ M of antioxidants was equal to 3.2% for hydroquinone and 1.5% for ascorbic acid (six consecutive measurements). Sensor-to-sensor repeatability was found to be 4.2 and 2.5%, respectively. All the sensors retain 80% of the sensitivity toward antioxidants (slope of calibration curve) during at least three months of storage in dry conditions.

### Real sample analysis

The electrochemical sensor containing 24 μmol of PTZANI deposited on bare screen-printed carbon electrode was tested in the determination of Fe(III) content in Ferrum Lek ® tablets containing dextran as stabilizer. The determination was performed by addition method after dissolution of the grinded tablets. With no pre-treatment, iron content was underestimated against standard samples of Fe(NO_3_)_3_ and nominal content of iron in the medication. To improve the recovery, the samples were heated with 1:1 nitric acid to destroy the complexes with dextran followed by neutralization of the solution. The results are presented in Table 2. As could be seen, the potentiometric sensor proposed showed satisfactory results of the determination of iron. Similar protocol can be used for estimation of biochemically available content of iron in foodstuffs and beverages.

**Table 2:** Validation of electrochemical sensor modified with 24 μmol of PTZANI on bare screen-printed electrode on the determination of iron in “Ferrum Lek” medication (six measurements).

№	«Added», M of iron	«Found», M of iron	Rel. Deviation, %
1	5 × 10^−4^	4.90 × 10^–4^	2.02
2	5 × 10^−4^	4.90 × 10^−4^	1.94
3	5 × 10^−4^	4.91 × 10^−4^	1.90

## Conclusion

We have studied the interaction of the synthesized PTZANI with emeraldine form of polyaniline and found aggregation of the mixture resulted in formation of the micron-sized particles. During the continuous ultrasonication, emeraldine particles are rearranged due to interaction between emeraldine and PTZANI with formation of stable self-organization associates. To the best of our knowledge, this is the first report on reorganization of emeraldine into nanofibrillar structures. According to the SEM data, nanofibers with an average diameter of about 30 nm dominate among other products of such interaction. The introduction of the PTZANI in the surface layer of the solid-contact potentiometric sensor on the platform of screen-printed carbon electrode was also performed. The potentiometric sensor exerted high reversibility of the redox reactions and ion-to-electron conductivity typical for emeraldine. Meanwhile the PTZANI based sensors showed advantages over those with polyaniline, i.e., more reversible pH-sensitivity of the potential, charge controlled response toward Fe(III) ions, ascorbic acid and hydroquinone. In all these cases, significant enhancement of the linear range of concentrations was achieved against polyaniline sensor. Addition of carbon black to the surface layer improved reversibility of the redox reactions as was shown by kinetic parameters of electron transfer obtained by constant current voltammetry with ferricyanide redox probe. Besides, potentiometric sensors with carbon black layer exerted higher reproducibility of the signal toward hydroquinone and Fe (III) ions and in the case of ascorbic acid improved sensitivity of the response and decreased in the detection limit by two orders of concentration magnitude. The potentiometric sensor covered with the PTZANI was tested on the example of the determination of iron in Ferrum Lek ® medication with satisfactory recovery and signal deviation less than 3%. Similar measurement protocols can be proposed for the assessment of biologically accessible amounts of iron and antioxidant capacity determination in food quality control.

## Supplementary information

Supplementary information
