# Supplementary material for: Phenyliminophenothiazine based self-organization of polyaniline nanowires and application as redox probe in electrochemical sensors
Source: Sci Rep. 2019 Jan 23;9:417. doi: 10.1038/s41598-018-36937-5 (PMC6344644; doi:10.1038/s41598-018-36937-5)
Supplement: Supplementary file 1 — Supplementary information [file 41598_2018_36937_MOESM1_ESM.pdf]

Phenyliminophenothiazine based self-organization of polyaniline nanowires and application as redox probe in electrochemical sensors

Alena I. Khadieva[1], Vladimir V. Gorbachuk[1], Gennady A. Evtugyn[1], Svetlana V. Belyakova[1], Ruslan R. Latypov[2], Sergey V. Drobyshev[3], Ivan I. Stoikov[1*]

[1]A.M. Butlerov Institute of Chemistry of Kazan Federal University, 420008, Kremlevskaya, 18, Kazan, 420008 Russian Federation,

[2]Institute of Physics of Kazan Federal University, 420008, Kremlevskaya, 18, Kazan, 420008 Russian Federation,

[3]Kazan National Research Technical University named after A. N. Tupolev – KAI, K. Marx Street, 10, Kazan, 420111, Russian Federation;

[*]Corresponding author. Tel.: +7-843-2337241; fax: +7-843-2752253; e-mail: Ivan.Stoikov@mail.ru

Electronic Supplementary Information

1. GC–MS
2. NMR

GC–MS

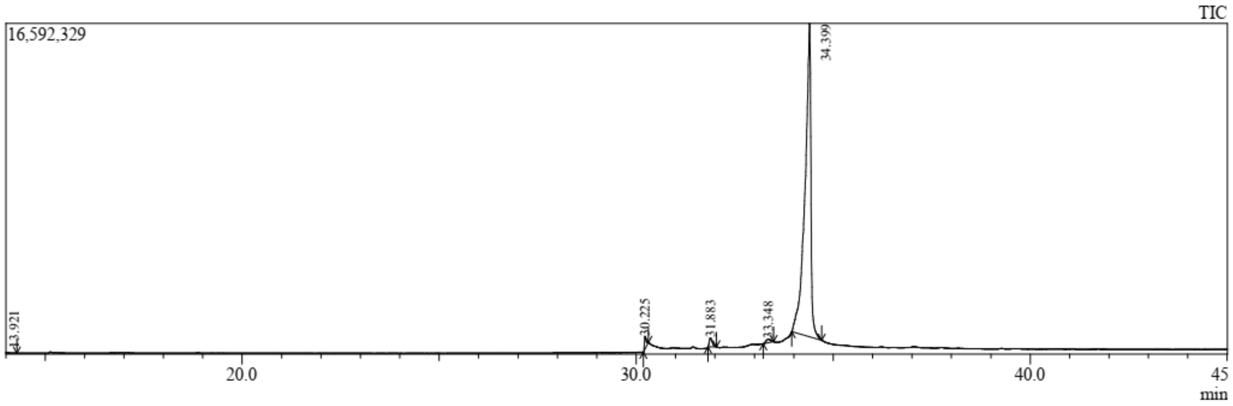

Peak Report TIC

Peak#	R.Time	Area%	Height%	A/H	Name
1	13.921	0.54	1.44	3.71	
2	30.225	1.12	3.67	3.02	
3	31.883	1.41	2.61	5.38	
4	33.348	0.84	1.11	7.55	
5	34.399	96.09	91.17	10.49	
		100.00	100.00		

Fig. S1. Gas chromatography - mass spectrometry (GC–MS) of (E)-*N*-phenyl-3H-**phenothiazin**-3-imine reaction mixture (Peak Report)

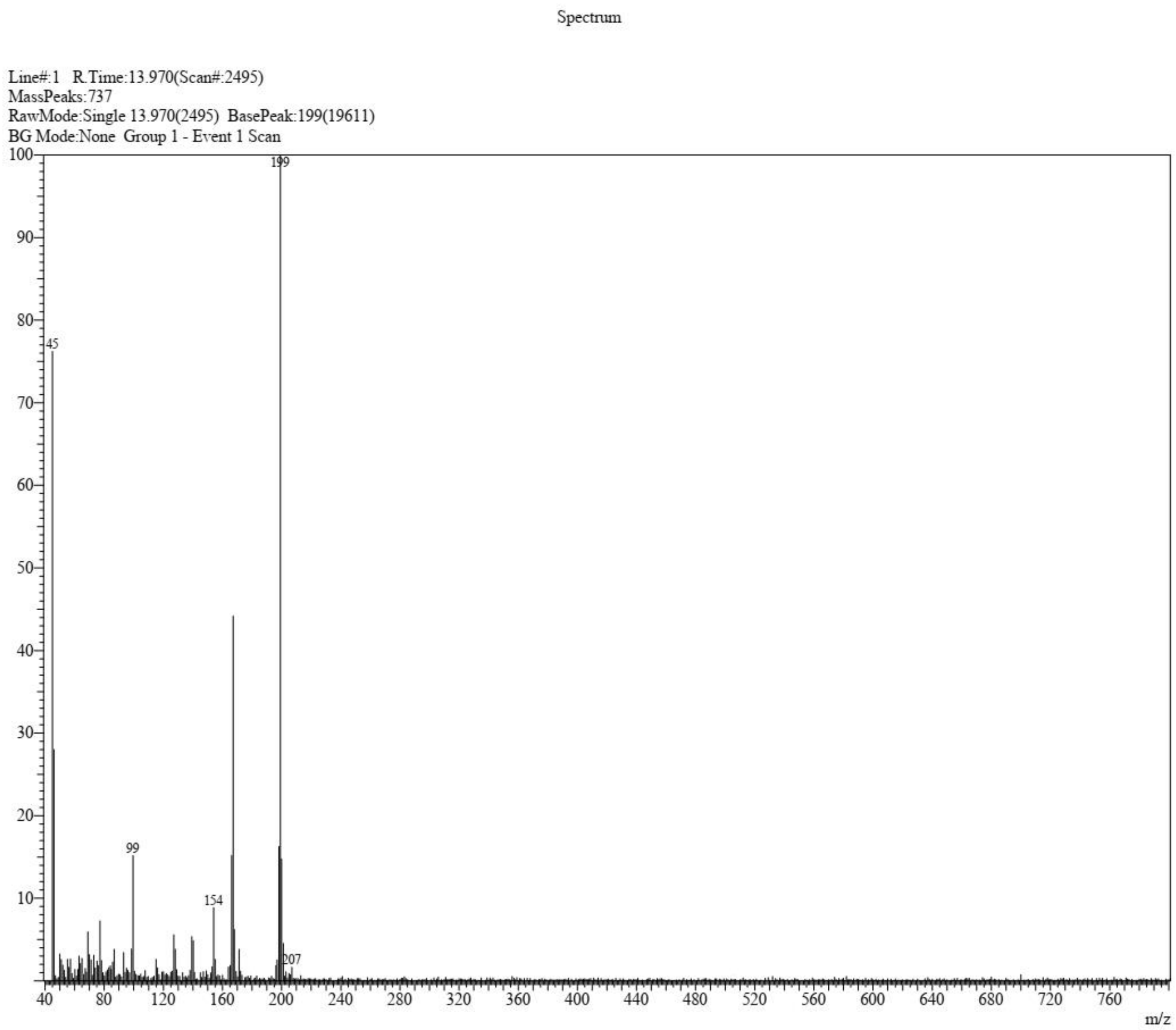

Fig. S2 Mass spectrum of (E)-*N*-phenyl-3H-phenothiazin-3-imine reaction mixture. Line 1 – phenothiazine.

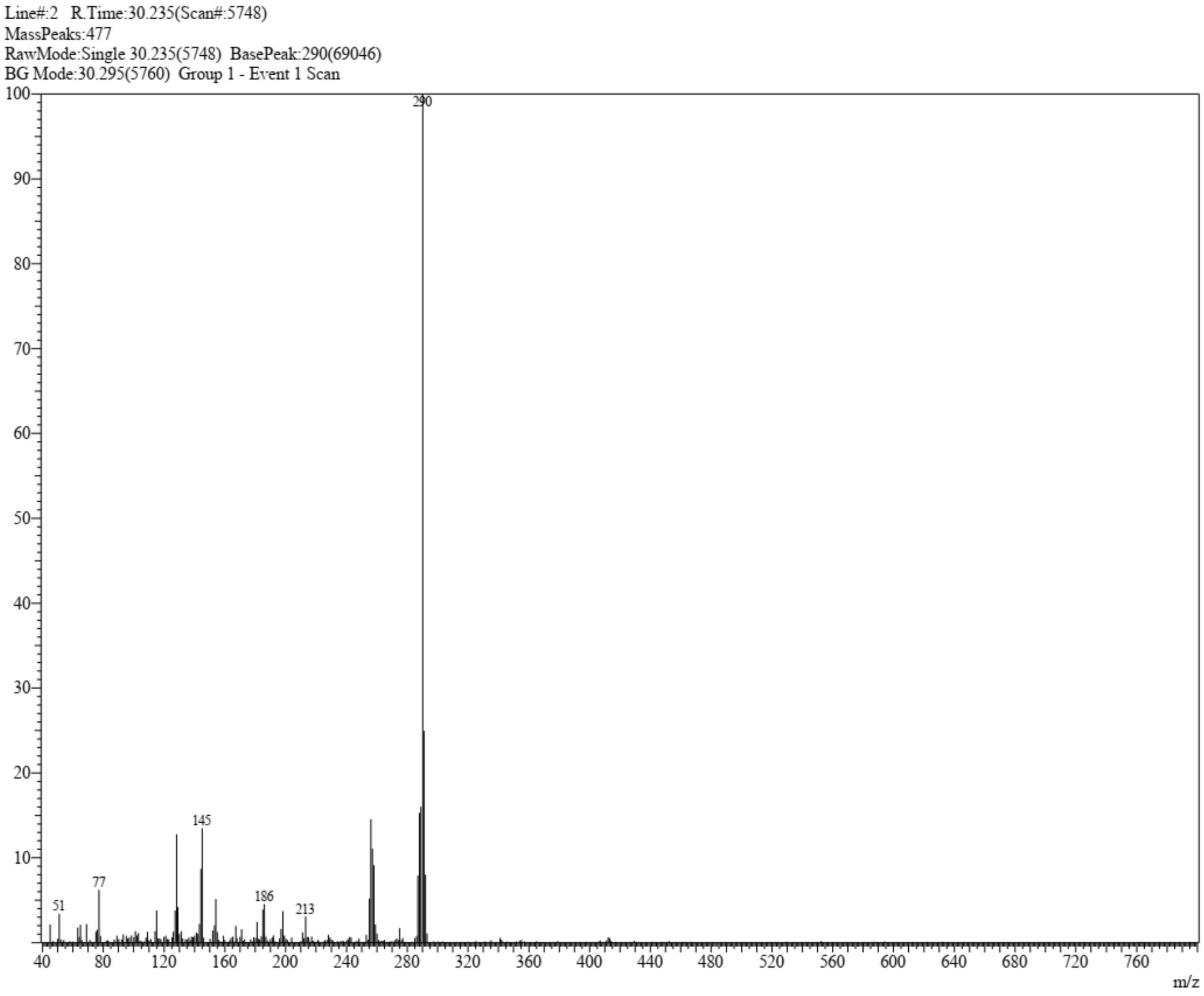

Fig. S3 Mass spectrum of (E)-*N*-phenyl-3H-phenothiazin-3-imine reaction mixture. Line 2 – (E)-*N*-phenyl-3H-phenothiazin-3-imine isomer

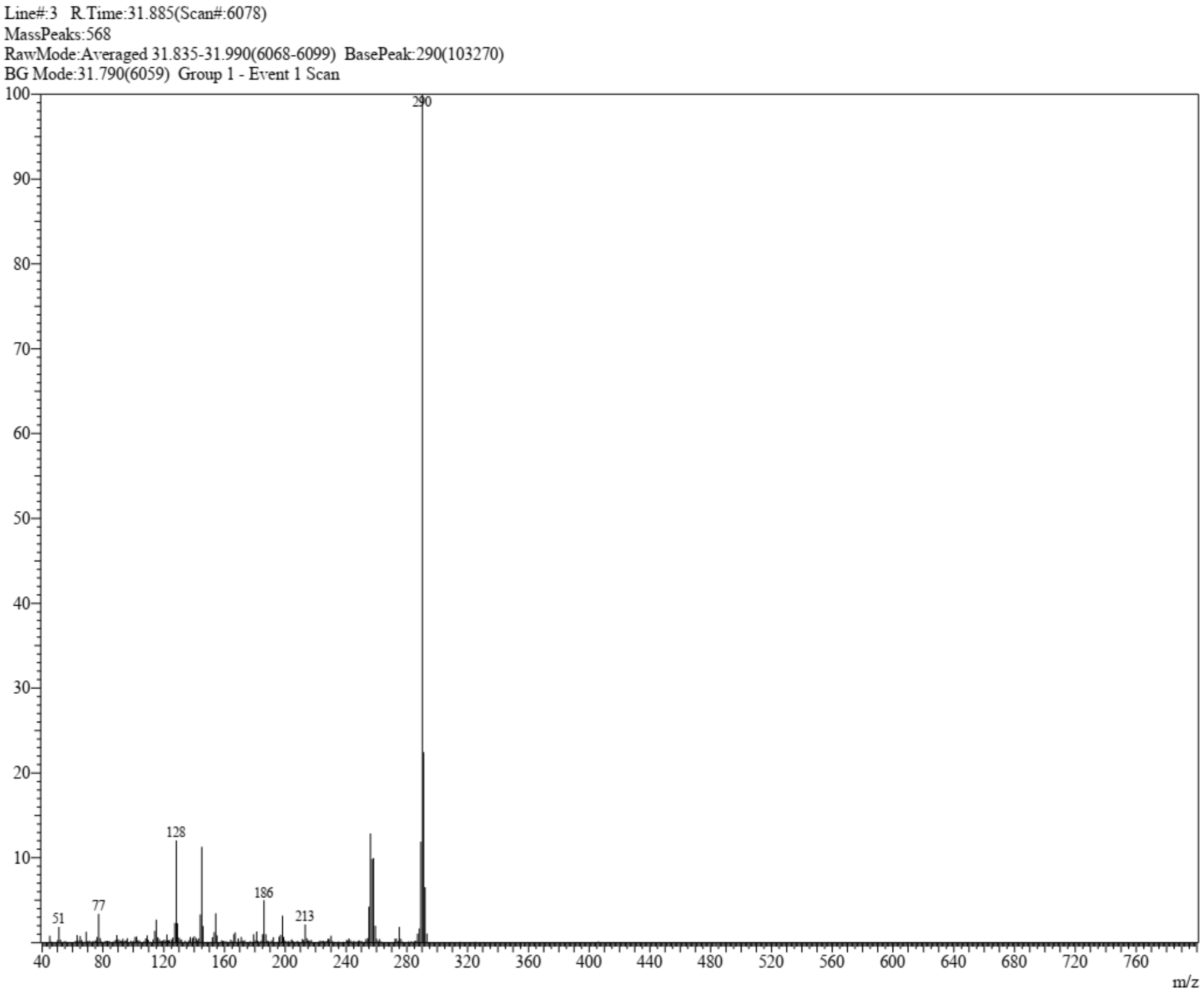

Fig. S4 Mass spectrum of (E)-*N*-phenyl-3H-phenothiazin-3-imine reaction mixture. Line 3 – (E)-*N*-phenyl-3H-phenothiazin-3-imine isomer

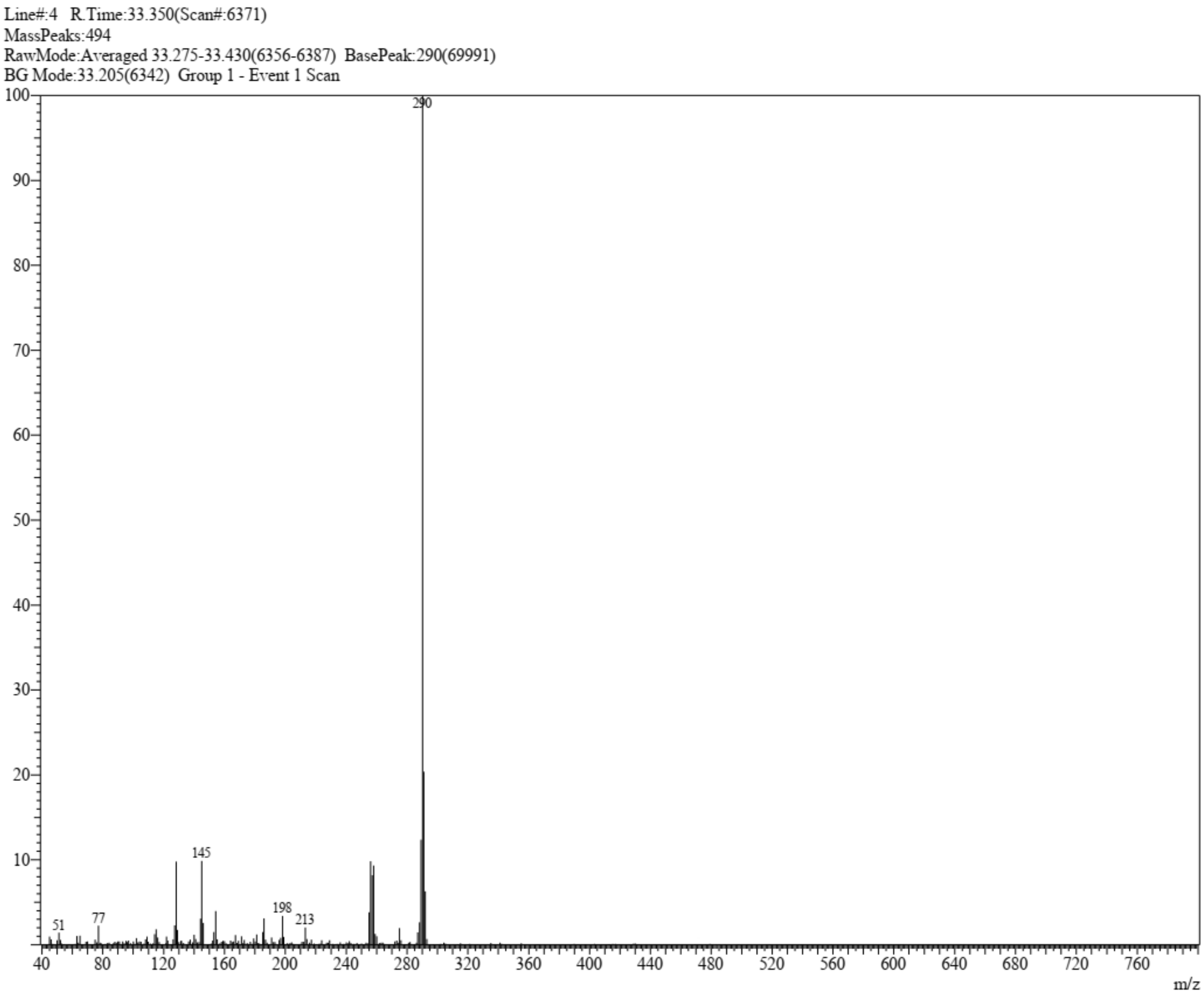

Fig. S5 Mass spectrum of (E)-*N*-phenyl-3H-phenothiazin-3-imine reaction mixture. Line 4 – (E)-*N*-phenyl-3H-phenothiazin-3-imine isomer

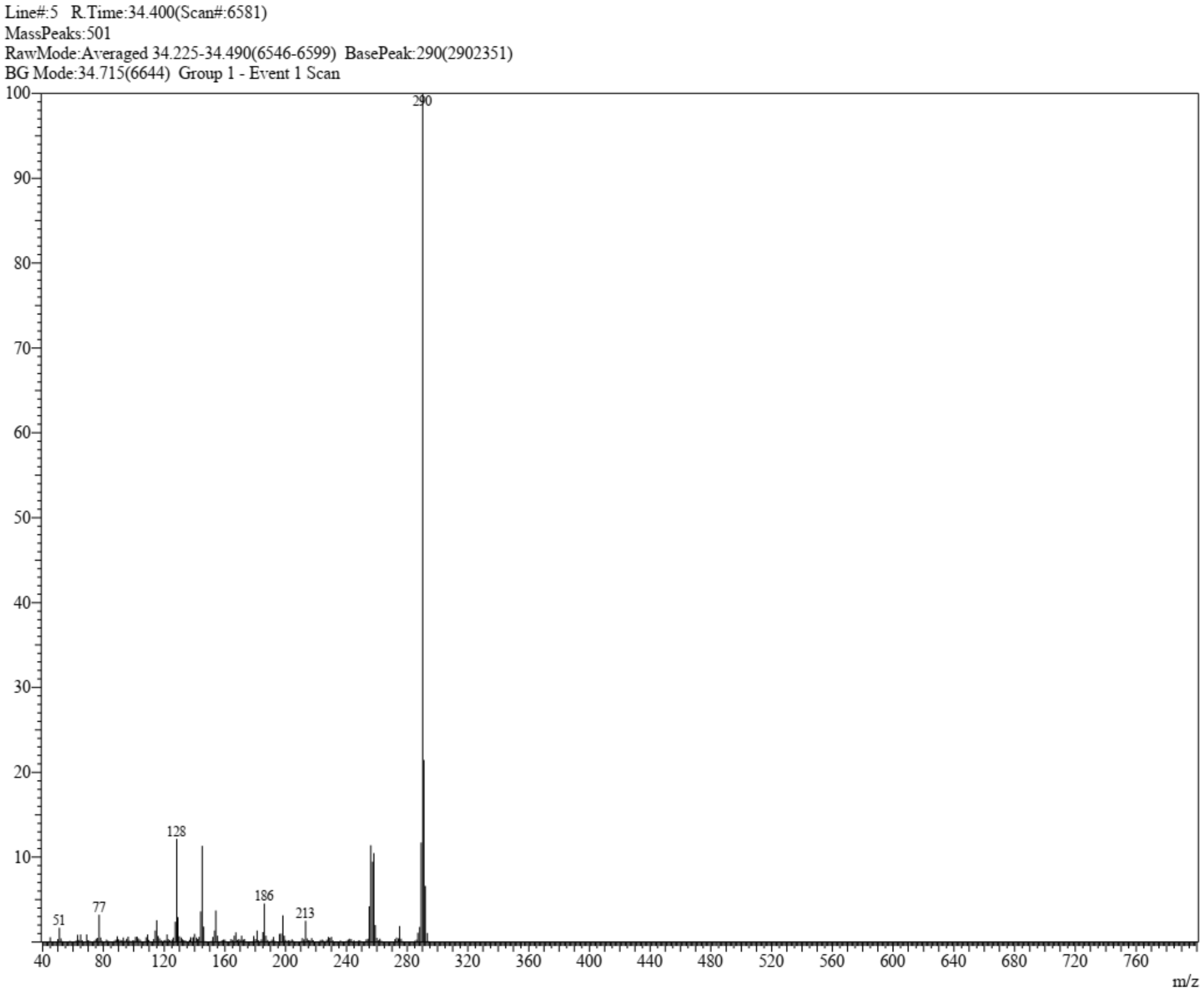

Fig. S6 Mass spectrum of (E)-*N*-phenyl-3H-phenothiazin-3-imine reaction mixture. Line 5 – (E)-*N*-phenyl-3H-phenothiazin-3-imine

NMR

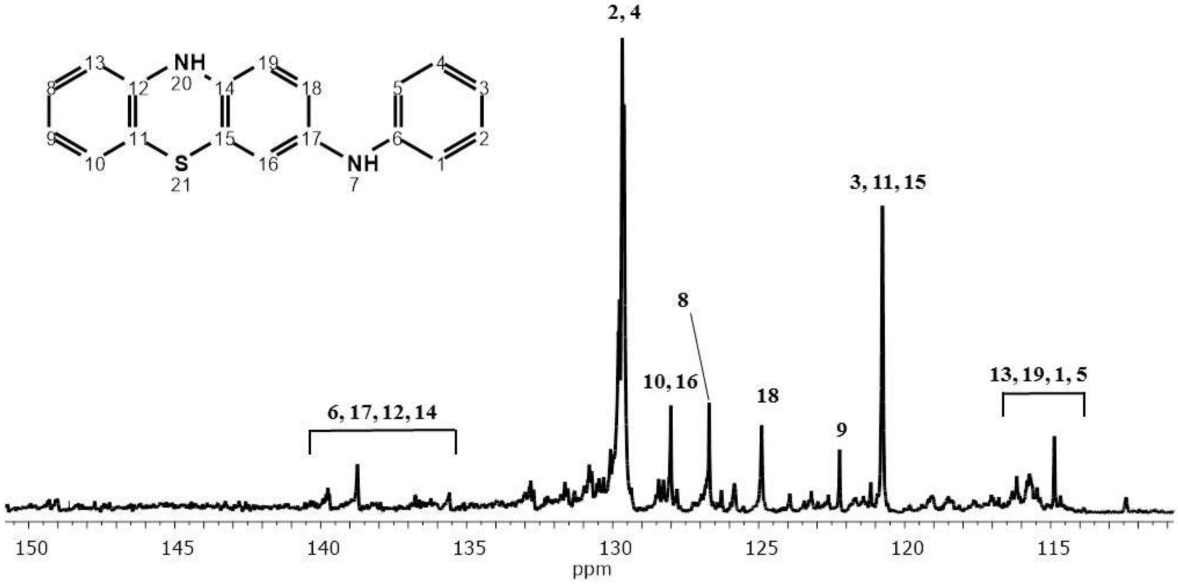

Fig. S7. ^{13}C NMR spectrum of (E)-*N*-phenyl-3H-phenothiazin-3-amine, reduced with phenylhydrazine $(CD_3)_2SO$, 100 MHz.

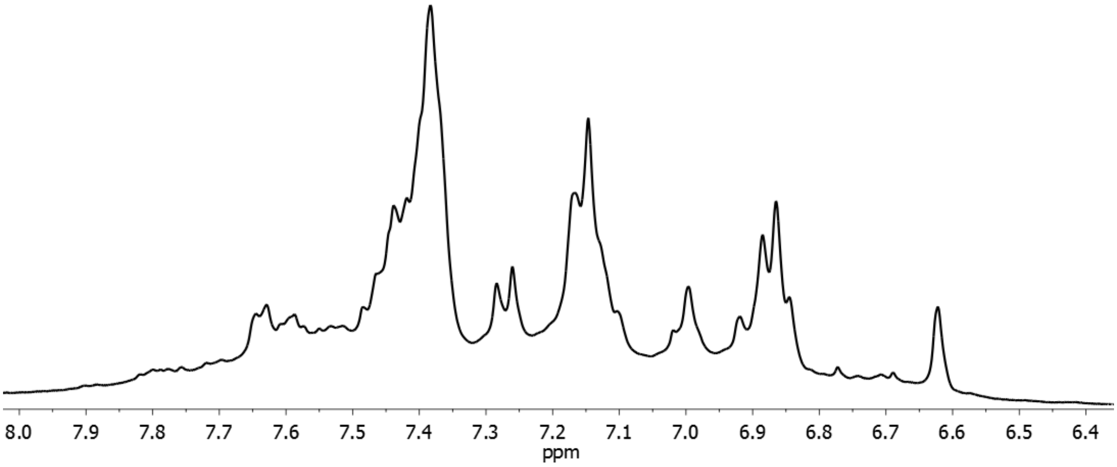

Fig. S8. ^{1}H NMR spectrum of (E)-*N*-phenyl-3H-phenothiazin-3-imine, $(CD_3)_2SO$, 400 MHz.

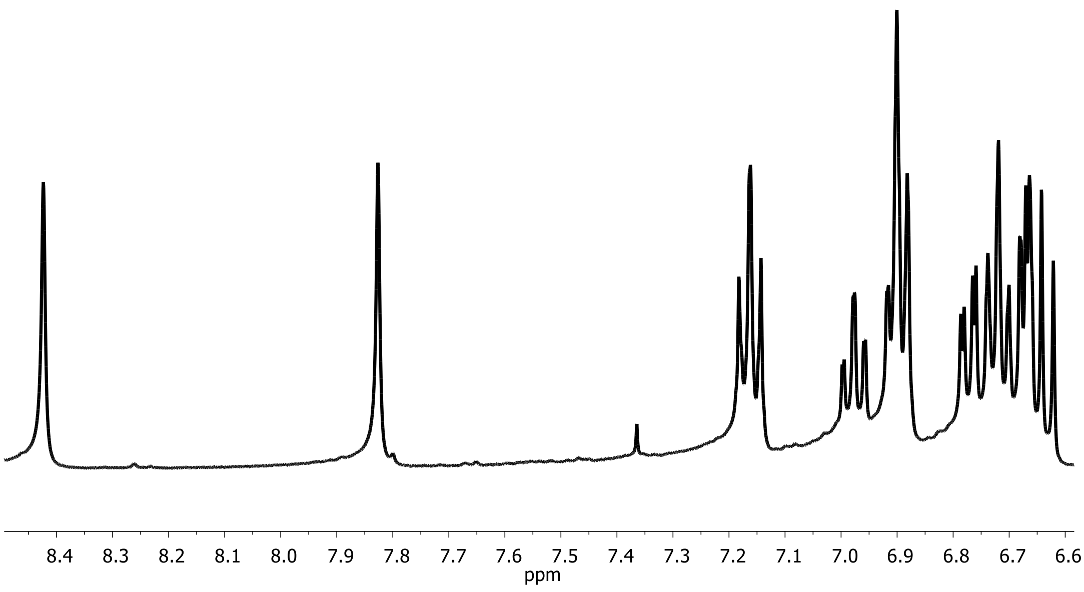

Fig. S9. ^{1}H NMR spectrum of (E)-*N*-phenyl-3H-phenothiazin-3-amine, reduced with phenylhydrazine, $(CD_3)_2SO$, 400 MHz.
